# The search for meaning in health care inquiries: introducing qualitative meaning analysis

**DOI:** 10.1080/17482631.2024.2382809

**Published:** 2024-07-25

**Authors:** Helena Dahlberg, Karin Dahlberg, Christopher Holmberg

**Affiliations:** a Department of health and care sciences, University of Gothenburg, Göteborg, Sweden; b Freelancer, Emeritus, Sjömarken, Sweden; c Department of psychotic disorders, Sahlgrenska University Hospital, Göteborg, Sweden

**Keywords:** Data generation, interviews, lifeworld, meaning analysis, nursing, qualitative methods

## Abstract

**Purpose:**

To describe how *Qualitative Meaning Analysis* (QMA), based on a lifeworld theoretical approach, can be made accessible to students and researchers not well-versed in the philosophy of science or qualitative research. Additionally, to demonstrate that it is a more rigorous approach than qualitative content analysis in guiding healthcare inquiries.

**Method:**

In recent years, qualitative approaches in nurse education and research have increasingly relied on various content analytical procedures. Liberated from clear philosophical underpinnings, they offer a seemingly pragmatic stance to nursing inquiries. However, by prioritizing ‘sorting content’ over the exploration of meaning, there’s a risk of adopting a mechanistic approach to qualitative analysis. This is problematic because we contend that the search for meaning lies at the heart of qualitative inquiry in nursing and healthcare research, dealing with existential phenomena surrounding health, illness, and care.

**Result:**

This paper explores the search for meaning in health care research, particularly in nursing, and introduces key epistemological aspects. It also discusses practical considerations to further familiarize and encourage the use of QMA in graduate nursing education and research.

**Conclusion:**

Qualitative inquiry with a focus on meaning is a powerful means when the intention is to develop person-centered care, and the relationship between the professionals and patients is in focus. Such an approach has the potential to illuminate existential suffering as well as innate health capacities in patients.

## 1. Background

We have observed that nursing students, including graduate and post-graduate students as well as PhD candidates, often struggle when introduced to qualitative data methods. Several factors likely contribute to this challenge. One significant factor we have identified is the difficulty in understanding the underlying philosophical aspects. Consequently, there is a tendency to favour instrumental approaches such as qualitative content analysis (QCA). This preference is seen not only among students but also among novice researchers in qualitative data methods, particularly those involved in higher education and supervising student theses. As Bengtsson (2016) argues, QCA does not align with a specific scientific discipline, and confusion pertaining to philosophical concepts and discussions is minimized. Some of the most used and cited qualitative data analytical approaches in nurse research and education are various content analytical approaches (Elo & Kyngäs, 2008; Graneheim & Lundman, 2004; Hsieh & Shannon, 2005), as shown in bibliometric analyses (Holmberg, 2024; Wang et al., 2022).

There are several concerns when it comes to QCA. Despite being labelled “content” analysis, the method is described to examine the meaning behind the content, using terms like “meaning units” and “interpretation” in studies (Elo & Kyngäs, 2008; Graneheim & Lundman, 2004). Proponents of QCA also acknowledge that there are no specific conceptions of what constitutes “meaning” in QCA (Bengtsson, 2016), yet state that the interpretation of a text involves “multiple meanings” (Graneheim & Lundman, 2004). The relationship between “content” and “meaning” is often neglected, and the understanding of interpretation tends to be closer to mundane language than to a scientific concept (H. Dahlberg & Dahlberg, 2019).

As we will show below, meaning often creeps in unnoticed while focusing on content. This implies that the results of a QCA do not demonstrate the thoroughness indicative of a scientific approach. Another concern is that, even though QCA aims to avoid philosophy for ease of practice, it is not without epistemological underpinnings. Content analysis originates from positivism, primarily recognized for creating a quantitative method in communication research (Berelson, 1952). While QCA has been adapted to handle qualitative data, some of the original guiding principles persist (Nicmanis, 2024). As noted by Roller (2019), these include isolating the analysis process from data collection and generation. Besides signalling that science is directed only towards analysis, such separation also contributes to a mechanized approach to qualitative analysis, limiting it to merely organizing experiences and sorting data, without revealing meaning.

Historically, there has been a general inclination towards instrumental approaches in nursing. The nursing profession, heavily influenced by medicine, has often adopted a mechanistic perspective. Patients have been viewed as a kind of ‘malfunctioning machines,’ and nursing research has followed a similar logic, identifying specific problems that could be neatly categorized based on their ‘content’ (Thomas & Pollio, 2002). We acknowledge that nursing has faced challenges in adapting to a medical paradigm that often overlooks the scientific robustness of qualitative methods. However, we contend that the solution does not lie in conducting qualitative research that is insufficiently equipped to address existential dilemmas in health, illness, and care while lacking sufficient rigour. There is a growing recognition that qualitative research plays a vital role in evidence formation, whether it pertains to specific treatment outcomes or health policy (Fisher & Hamer, 2020; Tucker et al., 2017).

Health care professionals’ attitudes towards qualitative research are not static and can be positively influenced by research educational initiatives (Ghirotto et al., 2020). There is also a long and rich tradition in nursing research, dating back to the 1970s and 1980s, which includes the use of phenomenology in qualitative studies by scholars such as Paterson, Zderad, Martinsen, and Benner (Norlyk et al., 2023). Drawing from our educational experiences in nursing, spanning all levels and including PhD programmes, we have developed an approach grounded in the phenomenological philosophy of science to teach Qualitative Meaning Analysis, which has been well-received by our students.

Firstly, we will explain the distinction between ‘meaning’ and ‘content’ by using continental philosophy of science and lifeworld theory. Subsequently, we will present QMA in a manner that renders it practical and beneficial for students at all levels.

## 2. Lifeworld theory

To articulate an approach to qualitative research that relates to meaning, we build upon lifeworld theory, which offers a capacity to illuminate, understand, and explain complex issues in health, illness, and nursing (K. Dahlberg et al., 2008). The lifeworld can best be understood as a world of perception, i.e., how we as humans experience and relate to everything and everyone that we encounter. In other words, lifeworld theory explores and describes the relationship between humans and our world, and how this relationship is one of meaning. We don’t choose to be or not to be part of the lifeworld, and likewise we don’t choose or not choose meaning. As humans, we are there already; we are part of the world and of meaning (Husserl, 1970; Merleau-Ponty, 1995). Paraphrasing Merleau-Ponty (1995), the notion that we can access the world merely by opening our eyes and dealing solely with content, while omitting meaning, is a delusion.

Exploring the realm of perception, Merleau-Ponty (1995) underscores the inevitability of engaging with meaning in our existence, asserting that we are “condemned to meaning” (xxii) just by being part of the world. He delves into the dual nature of the French term ‘meaning’ (*sens*). First, it is about *sense*, i.e., how a word or a sentence make sense to us, or how an event, a thing, or a human being makes sense. The declaration, “Now it makes sense to me!” encapsulates the active nature of perception, where we are consistently involved in making sense of our surroundings. Whether through sight, hearing, thinking, or any other perceptual form, there is always an object of focus, contributing to the continuous process of comprehension. Secondly, the French word *sense* carries another meaning – direction. When discussing the right direction in one’s life, the phrase *‘le bon sens*’ can be used. This meaning reflects a sense of purpose or orientation, something one strives towards but never fully attains. It signifies an ongoing development of meaning that evolves over time (H. Dahlberg & Dahlberg, 2020).

### 2.1. The lived body

In the health care and nursing fields, the lifeworld is explicitly expressed through the body, specifically the lived body. Merleau-Ponty’s (1995) significant contributions have been particularly influential in directing our focus towards the importance of embodiment whereby health, illness, and care can be explored and explained in ways that which complement the medical perspective. The establishment of a spatial as well as temporal framework provides us with stability and orientation as it is characterized by a mutually interdependent relationship between our body and our environment, involving relationships with others and co-creative processes. Among other aspects that are essential to health care professionals, our ability to stand upright is not solely attributed to skeletal mechanisms or the regulation of muscular tonus through the nervous system, but rather arises from our active engagement with the world. Without such engagement, one’s body loses its upright stance, becoming objectified and passive. Together with the temporal implications of movement in the past, present, and future, this emphasizes the dynamic interplay between the body, its surroundings, and the essential role of engagement in maintaining an upright posture.

The capacity we refer to as the “mobility of perspective” is revealed to be inherently symbolic. Rather than just engaging in movement and thereby embodying a sense, we also assimilate the already enacted sense of our familiar movements and express it through language, gestures, and cultural expressions such as creative endeavours. Human logos provides us with the ability to recognize meanings as such rather than just existing within them. The meaningful world to which our bodies respond encompasses meanings enacted by ourselves and others. As humans, we can explore and articulate these meanings in words. However, such meaning is present also in what QCA terms “content.”

The lifeworld, with its unique perceptions, experiences, meanings, and movements in time and space, is distinct and personal. Simultaneously, a person’s lifeworld is not an isolated ´bubble´; our way of existing is shared. The lifeworld possesses subjective, intersubjective, and objective structures, including language, cultural, and social patterns. When we observe each other, we realize our shared humanity, with common desires such as belonging and being loved in some ways. Building upon lifeworld theory, QMA includes all these insights.

## 3. Qualitative meaning analysis- how to do it?

The focus in QMA is the lifeworld. In health care, the focus is often on diagnosis and disease. However, in a lifeworld-led study, the interest is upon how the disease and/or care are perceived/experienced by the patients. Since the lifeworld also is about the everyday life, a QMA study includes an interest into how it is for the patients to live with health and illness as well as particular diagnoses in their everyday life, how they are experiencing this and how they can make sense of the situation, finding meaning in their everyday life, or relief from suffering, which may include work, leisure time, as well as family and friends. Having a lifeworld focus is to be interested in all kinds of existential issues, e.g., anxiety, sorrow, loneliness, as well as joy, dreams, and hopes. Such aspects of life are filled with meaning. As Merleau-Ponty (1995) shows, even the most manifest and apparent situations include meaning. Not least, it is of essential interest to understand the meaning of how these issues relate to health, illness, and care. So, how can such investigations be done, even by students and novice researchers in the health care area?

Qualitative Meaning Analysis (QMA) is at the core of phenomenological and hermeneutic research (H. Dahlberg & Dahlberg, 2020; K. Dahlberg et al., 2008). The strength of such research approaches is their robust foundation in continental philosophy. The continental philosophy provides all research with an appreciation of the difference between content and meaning, and, for example, what perception and interpretation mean, how one is open to a phenomenon in study and not ruled by one’s preunderstandings. However, as our teaching experiences show, these research approaches are often too dense for students and others who are novice to research. Our aim is therefore to present a QMA approach that is true to the epistemological groundwork but still uncomplicated/undemanding and straightforward to practice.^1^

Let us explain and illustrate how to do an investigation by an example. In the study that we borrowed from, the focus was on the lived experience of healthcare during pregnancy, birth, and three months after in women with type 1 diabetes mellitus (T1DM) (H. Dahlberg & Berg, 2020). Data came from lifeworld-oriented interviews with 10 women 27–37 years old, who had lived with diabetes between 14 and 26 years. Nine of them were being pregnant and giving birth to their first child. One woman had given birth once before.

### 3.1. Planning an investigation

Based on the lifeworld theory, a QMA investigation is characterized by some principles:

QMA is phenomenon oriented (H. Dahlberg & Dahlberg, 2020). Phenomenon orientation means that the inquiry is focusing upon something, i.e., a thing, an event, disease, or care, *as it is experienced by someone*. In our example, the phenomenon is *healthcare during pregnancy, birth, and three months after in women with type 1 diabetes mellitus*.

QMA demands an open attitude towards the phenomenon and our way of working in a study. As humans, we are continuously engaged with questions of meaning. Therefore, the challenge lies not in comprehending meaning per se, but rather in avoiding premature conclusions or hasty interpretations of meaning which are always present in our everyday situations (H. Dahlberg & Dahlberg, 2019). Engaging in openness means avoiding habitual and “simple” meaning, i.e., the meaning that we usually see that is common sense or obvious to us. The open attitude we are referring to is one of open eyes and ears, and an honest wish to be surprised. Such an open attitude is called “bridling” and refers to a stance where one slows down the movement of understanding in favour of a questioning, dwelling, and abiding attitude (K. Dahlberg et al., 2008).

In QMA, the open attitude begins already when planning the study and develops by initial questions about the phenomenon in focus; questions about what we already know about it and, in particular, what we do not know. All through the investigation one must repeatedly ask questions about one’s way of understanding. One slows down the pace, moves with and around the phenomenon, and is patiently waiting for it to show itself (H. Dahlberg, 2022).

### 3.2. Data generation choice of method sampling

In order to practice QMA and awaiting high-quality outcomes, high-quality data are needed. It is of principal significance to note that there are no QMA methods or lifeworld theoretical methods per se. On the contrary, all research methods can be of choice. The leading principle here is that it is the initial and preliminary understanding of the phenomenon together with the aim of the investigation that directs the choice of method.

#### 3.2.1. Interviews

Although all methods are viable options in QMA, interviews are considered a good choice based on their potential to illuminate, describe, or explain existential phenomena. Drawing from both previous research and our experience, interviewing is a method that can reveal lifeworld meaning and may, therefore, be a primary choice if participants are verbally capable.

In our example, individual interviews were used. All QMA interviews follow some main principles, which are exemplified by excerpts from the study:

1. QMA interviews are opened up by a question (or prompt) that leads on to the lived world of the interviewee: Can you tell me what an ordinary day is like for you?
2. After the ‘opening up’ dialogue, QMA interviews are structured by a few directing questions/prompts, preferably few enough to remember them by heart. One example of such a question in our exemplar study was: Could you remember a meeting where you had experienced really good care? Other questions were: How was it for you to be pregnant/give birth? Tell me about the professionals and the hospital care! How was it to come home again?
3. Most important in a QMA approach is to ask many following-up questions/prompts. When a woman in the study described how really good it was to meet “her” physician and nurses, the expression was followed up by a question: “In what way was it good?”

Other examples of following-ups/prompts are: Tell me more about that! Can you give me an/another example? Is there another time when you felt like that/or differently?

These examples point to the openness of the interviewer that s/he is present in the interview situation. In every moment of the interview, it is crucial to be aware of what, how and when the interviewees say about the phenomenon. In everyday conversations, we take for granted that we understand what the other conveys. In interview, however, we cannot take this for granted, but have to think that we *don’t know* what the other is revealing. It’s better to listen actively to the interviewee than to find the next good question! In such a way, interviews have opportunities to provide any investigation with experiential depth, which for both the interviewer and interviewee may be new and previously unreflected.

Also, in everyday conversation, we usually don’t like too much silence. In QMA interviews, there may very well be silent spaces where the interviewee may find enough space and new ways to describe something that is difficult to speak about or not thought about before.

#### 3.2.2. Narratives or stories

Another option to gain verbal data is to ask for narratives or stories about lived experiences of something that is in focus. Let us say that we plan for an investigation on loneliness, as it is experienced by teenagers. They can then be asked to write a story about a time when they felt lonely. The study could also include the opposite, i.e., stories about a time when they did not feel lonely. Either way they should be asked to be as descriptive and detailed as possible, e.g., describe the contexts, the presence of other people and their roles, what people said or did not say, what characterized the experiences, etc.

#### 3.2.3. Other means

Participant observation may be a choice if the participants are not verbally capable, e.g., if they are suffering from aphasia, if it is about children not yet having developed language skills, or immigrants with foreign languages that don’t work in the study (K. Dahlberg et al., 2008). Observation is also an important method if the study focuses upon a particular situation, cultural aspects etcetera. An investigation can also benefit from inclusion of other method means such as using motif cards, or including drawing or painting, as well as drama (Kirkham et al., 2015). QMA in student inquiries can also be based on published biographic texts, where minimal efforts are needed to provide the investigation with data for the analysis.

When data generation methods are decided upon, it is time to invite participants (or find biographical texts) to the study. Explicit sampling criteria are central to all kinds of inquiry. It is essential to purposefully select participants who possess lived experiences relevant to the phenomenon under study. The focus should be on generating rich data that explores a range of experiences related to the phenomenon in focus (H. Dahlberg & Dahlberg, 2020; Giorgi, 1997). As a lifeworld-led inquiry, QMA demands from data to show variation, e.g., in gender, ages, rural/urban, depending on what kind of phenomenon that is in focus. It is important to be open to and question the search for variation so that it is not based only on the investigator’s presuppositions (Norlyk & Harder, 2010; Vagle, 2014).

### 3.3. Data analysis

QMA is characterized by a whole – parts – whole approach. One begins with the data text as a whole, which is read repeatedly to be familiar. The search for meaning begins with putting the whole text into parts with each one showing some meaning. After additional rounds of reading, one may see that there are clusters of meanings, i.e., meanings that show similarities in meaning and those that are different from others. In such a way, one can form clusters of meanings that show the constituents/elements that together make up for the phenomenon, and – preferably – describe it in a new way. From the parts, a new whole is to be presented.

The open, bridled attitude that is part of QMA is practiced during the analysis, mainly by asking questions to the text, such as: “What is said?” “How it is said?” or “What is the meaning of this?”, “Is this a meaning that belongs to the phenomenon?”, “Does it mean something else than what we thought at a first look?”, “Are there meanings that surprise us?” It is important not to rush through the analysis but to instead dwell with the data, being open to unsuspected and new meanings. If the thorough work of going back and forth in the data and asking questions of meaning works well, the analysis will reveal meanings that run all through the data and thus say something interesting and new about the phenomenon in focus.

Openness to unexpected meanings includes an ability to also dwell *in-between* possible meanings. It is like when one wants to convey something and is trying to find a particular word, a word that is not coming out at the moment, but is “on the tip of my tongue” (H. Dahlberg, 2022). There are some important meanings to be found, they are *right there*, but one doesn’t have the right expression yet. In such situation, one must actively wait for the words to come, asking questions, trying different words, sometimes alone and sometimes together with colleagues.

Let us explain and illustrate the search for meaning by returning to our study exemplar (H. Dahlberg & Berg, 2020). Due to very good interviews, built up by only a few directing questions/prompts and really many following-up questions/prompts, there was high-quality data to work with. The researchers noted many meaningful excerpts from the interviews and discussed how they could find meaning in them. One example of a preliminary meaning that came up was that instead of perceiving oneself as a capable woman, ***there seemed to be a strong focus on diabetes as a disease, during pregnancy, birth, and after birth***. The meaning is built up by excerpts such as, “I’m never so much a diabetic, I’m never so ill, as when I am pregnant.” “ … for me, the technical equipment’s are double-edged, they helped me very much, but they are also a reminder … hello, you are sick! Don’t forget that.”

**Table:** 

**Box 1. Example to illustrate an investigation with a lifeworld focus (H. Dahlberg & Berg, 2020)**. Pregnancy, childbirth, and the initial months of motherhood, poses difficult challenges for women diagnosed with type 1 diabetes mellitus (T1DM). The study was therefore conducted to describe the lived experience of health care in women with T1DM, during pregnancy, labor, birth, and up to 12 weeks after birth. Data came from lifeworld-oriented interviews with 10 women 27–37 years old, who had lived with diabetes between 14 and 26 years. Nine of them were being pregnant and giving birth to their first child. One woman had given birth once before. Interviews were analyzed by focusing on the meanings of the study phenomenon.

**Table:** 

Criteria	Author Initials
Made substantial contributions to conception and design, or acquisition of data, or analysis and interpretation of data.	HD, KD, and CH
Involved in drafting the manuscript or revising it critically for important intellectual content.	HD, KD, and CH
Given final approval of the version to be published. Each author should have participated sufficiently in the work to take public responsibility for appropriate portions of the content.	HD, KD, and CH
Agreed to be accountable for all aspects of the work in ensuring that questions related to the accuracy or integrity of any part of the work are appropriately investigated and resolved.	HD, KD, and CH

It was also obvious that the women regularly were monitoring the disease in order to prevent from negative consequences for the child. An observed meaning in this context was about ***constant control and focus on risks***. This meaning was illuminated by excerpts such as “I have never felt so controlled as when I was pregnant”. There were discussions about how the illness was more apparent when the women were pregnant, compared to “just” having diabetes.

Another preliminary meaning was focusing on ***the women’s responsibility of the child*** and its health and was built from excerpts such as, “ … it was not only my own body, but I had the responsibility also for the other being. I alone had the responsibility”. “ … it was a ‘guilt-responsibility’, it’s not a word but … this responsibility belonged to a sort of guilt: if I don’t manage my diabetes and then he [the baby] could be ill and then I will carry this yoke for the rest of my life”.

Eventually, the researchers found out that these preliminary meanings could be described by one theme of meaning: C**onstant monitoring highlights the illness, the risks, and the responsibility**. With these examples in mind, we now want to illuminate three different ways of describing the results of the analysis, all of which are meaning oriented. These ways vary in their analytical depth and the extent to which they incorporate external data (e.g., theory). Consequently, the most appropriate way should be determined by considering both the quality of the data available and the experience and capability of the person(s) conducting the analysis.

1. The most common form of QMA is directed towards some *themes of meaning*. The themes present clusters of meanings that data convey about the phenomenon. Different from most other forms of thematic analysis, these themes of meaning originate in the lifeworld and are consequently illuminated with epistemological support from the lifeworld theory.
2. If the data are rich enough, it is also possible to deepen the analysis in a way that results in a *full structure of meaning*. The themes of meaning are then bound together by a presentation of essential meanings that characterize the phenomenon in study. These two ways of completing the analysis include only the empirical data generated within the study.
3. A third option is to include *external data*, e.g., theory, in the analysis. The reason for including theory in QMA is that the empirical analysis shows too many blind spots, i.e., there are too many questions that cannot be answered by data only, and then theory (e.g., philosophical, health, nursing, cultural, sociological) or e.g., previously reported research can be included in the analysis (H. Dahlberg & Dahlberg, 2020).

#### 3.3.1. Describing themes of meaning

When all the data in the exemplar study exemplar (H. Dahlberg & Berg, 2020) were thoroughly worked through, the research team could present four themes of meaning:

##### 3.3.1.1. Constant monitoring highlights the illness, the risks, and the responsibility

The interviews conveyed how the women were constantly monitoring their illness, e.g., the blood sugar level, blood pressure, etc., and its eventual consequences for the child. The focus upon monitoring and their results left the women with feelings of guilt, e.g., that their way of relating to their health might be harmful to their baby. As an example, one woman expressed that whereas before she related to her illness as a slight impairment, like having to wear glasses, she had now come to understand it as critical, since her blood glucose levels would immediately affect her unborn baby. This unceasing control was described in words such as “hysterical” and “exhausting” and as “a fulltime job”. “I’m never so much a diabetic, I’m never so ill, as when I am pregnant. How can I say … you embrace your disease much more. It’s not that the illness gets more ill but rather … [that] I have entered a place where it’s my job to keep my blood glucose. That’s my job and it’s a full-time job”

###### 3.3.1.2. Needing professional support to lift the responsibility and ease the guilt

The women’s experiences of both illness and the associated risks acknowledged their need of care and set specific requirements on the healthcare that they received. For example, they wanted to hear that they were doing a good job that they were doing all that could be expected of them in order to be able to relax and to let go of anxiety. Such affirmation was crucial, as being pregnant implied an underlying feeling of guilt for putting the child at risk. “It usually feels very nice, because I have very high demands on myself and when I’m there [at the antenatal clinic] I always get confirmation that I’m doing a good job, they tell me to relax”.

###### 3.7. Needing to be met as a unique person

Among these women, there was a prominent need to be met, seen, and understood as a unique person. They appreciated it when the healthcare professionals expressed genuine concern for them, as opposed to “just doing their job”. Such a relationship was described as personal and friendly and was, for example, experienced when the professionals took time to visit the women at the postnatal ward or expressed joy when seeing the newborn baby. One woman had experienced such a genuine relationship with her diabetes nurse and described it in the following way: “She is someone that I could dare to call almost in the middle of night, if I were to need it.”

#### 3.8. Being lost in the healthcare system

As pregnancy, childbirth, and early motherhood were a new situation for the women, they needed to know and understand what was happening in care and why. If that was not the case, a feeling of being lost was prominent. To feel lost was the opposite of feeling taken care of, and this feeling arose when the information was not clear or concise enough for them to understand the situation, or if they could not find their way in the health care system, e.g., due to the specializations and several professionals to relate to. “So, I felt like I couldn’t trust them, and then, of course, I felt lonely. It’s hard when you can’t trust the healthcare staff. It does something to you, because you’re already in a vulnerable position and then you just want to be able to let go of control … And I couldn’t”

## 3.3.2. Describing a full structure of meaning

In the study that we use as an exemplar, they achieved really good data and could therefore present a full meaning structure, including both the themes of meaning presented above and essential meanings that are comprehensive and include all the themes. The advantage of presenting a full structure of meaning is that the themes of meaning, instead of just being lined-up, they are now explicitly related to each other and bound together by the description of essential meanings. In such way, the study result more clearly defines the phenomenon. Another advantage is that with essential meanings, the results are easier to transform to other situations and contexts.

In the exemplar study, the phenomenon, *the lived experience of healthcare during pregnancy, birth, and three months after in women with type 1 diabetes mellitus*, can be essentially and in short described as follows: The lived experience of having T1DM, being pregnant and giving birth, and of becoming a mother the first months after birth, appears as a period in which the woman to an even higher degree than usual must relate to her diabetes disease and the risks and responsibility that comes with it. The process from pregnancy to after birth is characterized by a constant monitoring, performed by the woman herself as well as by the healthcare professionals. This highlights the diabetes illness, its risks, and the responsibility associated with it, before, during, and after the birth of the child. It gives rise to a wish to share the burden of risks and responsibilities with healthcare professionals, and to get support from them. For this to be possible, the women need to be in a personal and friendly relationship with the healthcare professionals, a relationship that can offer individual and sensitive support and ease feelings of responsibility and guilt.

## 3.3.3. Including theory in the analysis

In the exemplar study, theory was not included in the analysis. However, we can still use this example and see how theory could be included.

One reason for the women not being met and seen as persons was that the healthcare professionals were experienced as being blinded by “medical blinkers” and thus unable to see the situation from the woman’s perspective. This led to a failure to meet, see, and talk with the women in the way that they needed. The example of a woman who, during the ultrasound, was informed that the doctor could not find the foetal heart illustrates this. The doctor was so absorbed in her/his own medical perspective, including the task of identifying all the different organs during the ultrasound, that s/he forgot to communicate in a professional and human way with the woman. The reason why a doctor speaks about a missing heart without realizing how this is conceived by the patient is probably not malevolence, but more likely unawareness and a difficulty to see beyond one’s own perspective.

Now, one way to further understand this sort of communication breakdown is by describing the two different “voices” employed by patients and healthcare professionals in situations like this. Mishler (1984), and later Barry et al. (2001) have described how healthcare professionals sometimes use “the voice of medicine” while patients speak with “the voice of the lifeworld”. While patients speak in everyday language about their concerns, where their concerns and symptoms are tied up with their existence, healthcare professionals stay rigidly inside a biomedical format, a format that is de-contextualized and impersonal. Already in the analysis, the findings of Mishler and Barry et al. could be included and related to the experiences of “medical blinkers” in the empirical data. In such analysis, results could be clearer about how it is to patients who are more or less reduced to a disease or diagnoses, instead of being personally met. It would also be possible to include publications of lifeworld-led care, emphasizing the idea of the lifeworld as a meaningful, interconnected world, which might give more suggestions as to what it is in health care to meet a patient as a unique person, as a human being focusing on health rather than disease (K. Dahlberg et al., 2009; Todres et al., 2007).

## 4. Discussion and conclusions

In this paper, we aim to explain and demonstrate how Qualitative Meaning Analysis (QMA) can be applied and evaluated in a manner that is accessible to individuals with limited experience in qualitative inquiry. Our intention has been to promote the use of qualitative data analytical methods that emphasize *meaning* over *content*. Drawing on our experiences as researchers and lecturers in higher education nursing and healthcare institutes, we recognize the tendency of students to opt for approaches that provide “recipe book-style” descriptions of analytical procedures, following a step-by-step format. To counter these tendencies, which we argue do not benefit healthcare research and education in general, and particularly in nursing, our goal has been to offer an alternative approach. We do so by outlining accessible theoretical and practical insights into how a lifeworld-led qualitative inquiry, focusing on meaning, can be conducted.

In addressing these objectives, this paper also engages with common methodological issues found in published nursing literature, highlighted by Norlyk and Harder (2010). One such issue is the oversight of the researcher’s role of openness, resulting in instances where the authors’ presuppositions impact the study, for example, by using narrowly focused interview questions. Maintaining an open attitude is crucial because familiarity with certain aspects may lead to seeing them through familiar eyes, preventing us from discovering novel aspects. Investigators need to carefully monitor their evolving understanding to ensure it develops deliberately. In QMA, this practice involves being present, questioning one’s understanding of a phenomenon, rather than taking it for granted, with the ultimate goal of opening up numerous possibilities for understanding (H. Dahlberg & Dahlberg, 2020).

QMA also addresses the prevalent belief about a methodological gap, rooted in epistemology, between descriptive and interpretative analysis (Lopez & Willis, 2004; Matua & Van Der Wal, 2015). As discussed elsewhere (H. Dahlberg & Dahlberg, 2020), there is a misinterpretation of the original philosophy’s intent behind both “description” and “interpretation.” “description” in a phenomenological sense aims to reveal the way things come to be as they are, to unveil meanings, which go beyond recording individual experiences (as in content analysis). Consequently, “interpretation” is not the opposite of “description”; there are no fundamental differences between describing meanings and interpreting human actions through language or other forms of human activity. Thus, an underlying assumption is that all living beings orient themselves both towards *an objective world* and towards an *environment organized in alignment with their preferences and vital goals*. Therefore, achieving a comprehensive understanding of meaning involves presenting intentional meaning through analysis of only empirical data *and* by conducting analysis including external data, e.g., theory or conceptional frameworks. QMA enables this by emphasizing the importance of maintaining an open and reflective approach to analysis grounded in lifeworld theory.

Similarly, Qualitative Meaning Analysis (QMA) addresses the common misconception concerning the characterization of variation in qualitative studies, as pointed out by Norlyk and Harder (2010). Variation is often defined through empirical research criteria, such as sample size, age, and gender distributions. However, these criteria are more aligned with quantitative research than with the essence of qualitative research. Even though age and, e.g., gender, as well as education and work data can be of importance, QMA emphasizes the need to shift the focus towards fostering rich and nuanced descriptions of experiences from participants. The true essence of variation in qualitative studies lies not in demographic factors but in the depth and diversity of individuals’ lived experiences. As highlighted by K. Dahlberg (2006), when descriptions are complex and multifaceted, they unveil a wealth of variations inherent in the phenomenon under investigation. Thus, QMA encourages investigators to prioritize the exploration of the richness within participants’ experiences, for a more profound and authentic understanding of the studied phenomena.

Meaning holds a central position in qualitative research as it is a core attempt of qualitative researchers to make sense of, or interpret, phenomena in terms of the meanings people bring to them (Aspers & Corte, 2019). To emphasize this focus and to help guide students and novice researchers, we have named the approach presented in this paper “Qualitative Meaning Analysis” (QMA). The chosen name, Qualitative Meaning Analysis (QMA), also serves to highlight the contrast with Qualitative Content Analysis (QCA), an approach that lacks a clear definition of the relationship between “content” and “meaning” or the role of interpretation (H. Dahlberg & Dahlberg, 2019). In QCA, proponents argue that interpreting a text involves recognizing “multiple meanings” (Graneheim & Lundman, 2004), and the absence of a clear definition is considered a strength for its flexibility (Bengtsson, 2016). However, neglecting to define the concept of meaning not only diminishes the significance of qualitative data analysis, but it also bears methodological implications. It may lead to a mechanistic application of methodology – essentially, replicating a methodological technique without a careful analysis of its relevance to the studied phenomena, particularly when meaning is assumed.

This is why we in this paper about data analysis also stressed the importance of data collection and data generation. The relationship between the participant and the researcher assumes a pivotal role in QMA, not only in ensuring the integrity of collected data but also during the data collection process. This illustrates another key difference between QMA and QCA pertaining to the participant–investigator relationship (Roller, 2019). In contrast to QMA, where the participant–investigator relationship is acknowledged to be important for both data collection and the integrity of the collected data, the QCA investigator maintains an apparent distance from study participants. Instead, the focus is almost solely on content obtained at a prior point in time. Consequently, potential bias originating from the participant–investigator relationship becomes inherent in the content. However, accessing subjectivity is crucial for understanding the uniqueness of participants. This approach enables the retrieval of people’s experiences, even if incomplete, and meanings as valid sources, given their certainty to the individuals who live them. The qualitative analysis of a phenomenon should extend beyond mere organization and classification of individuals’ experiences and meanings.

### 4.1. Implications for nursing

In this paper, we demonstrate how health care inquiries, especially for beginners, can benefit from a lifeworld theoretical approach to qualitative analysis that seeks meaning. An intention has been to encourage students and novice researchers within nursing and health care to adopt a lifeworld approach in their qualitative inquiries through the use of QMA. In doing so, we hope to foster research practices that embrace greater openness and sensitivity towards the phenomenon under study, including existential issues.

The lifeworld theory, guiding QMA, can also form the basis for clinical work. Nursing, defined as a profession of caring, is guided by caring science theory (Todres et al., 2007). In this context, health is not merely the absence of illness but includes well-being, as perceived by the patient. While medical science focuses on the biological aspects of disease or illness, caring science emphasizes the patient’s health, existence, and life influenced by illness. Therefore, the patient cannot be isolated from life’s broader context in caring situations. Nursing aims to understand and support the patient’s lived experiences, fostering health through a nurse–patient relationship involving caring communication and nonverbal presence. Both the patient and the nurse contribute expertise from different perspectives: the patient as an expert on personal and individual existence, and the nurse drawing from professional knowledge and experience. An important way to concretely practice this philosophy is to document the patient’s narrative, as it is within this narrative that the person’s resources and obstacles can be identified (Ekman et al., 2011). Such practice facilitates the understanding of patients’ care-related experiences in relation to their lifeworld, viewed as a lived body embodying all aspects of the human experience. Articulating a care philosophy aligned with these principles allows us to acknowledge the importance of tailoring care approaches to the individual’s lifeworld. This ensures that therapies and treatments are not only contextually relevant but also sensitive to the person’s unique experiences.

Just as in clinical work, it is equally important to emphasize the importance of an open and reflective approach to understanding the lifeworld of others in nurse education and research. To consider the individual’s lifeworld, we need to recognize that well-being encompasses physical, mental, and existential dimensions. As patients undergo changes in their familiar existence, caring science, alongside other sciences, becomes crucial to address multidimensional suffering. While the patient–nurse relationship is inherently dynamic, patients, due to illness, may struggle to articulate their experiences. Caring thus requires the nurse’s openness and engagement with the patient’s expressions, combining professional knowledge with empathic insights. These aspects need to be met in education. A concrete example of this, is lifeworld-led education, which we illustrate with Hörberg et al. (2019) article. They describe a combination of different learning activities which all aim to facilitate a deeper and expanded understanding of human vulnerability as a resource for care.

### 4.2. Phenomenology, nursing, and a way forward

Qualitative analysis is valuable in all health care research, particularly in nursing. Nursing is often intrinsically meaningful due to the direct patient care and the potential impact on patient outcomes. However, persistent confusion surrounding content and meaning in content analysis, coupled with an overreliance on standardized methodologies, has yielded unfavourable results. Despite some supportive initiatives to facilitate the learning of qualitative methods, this method of dealing with methodology appears to be a shortcut that may lead to a lengthy route with an unknown goal. We argue that understanding and engaging with meaning are critical aspects of qualitative research. Based on the lifeworld theory, we have demonstrated how students and novice researchers can effectively handle Qualitative Meaning Analysis, shedding light on, describing, and explaining existential issues in all health care, particularly in nursing.

The depth and diversity of individuals’ lived experiences are complex and multifaceted, making descriptions prone to appearing abstract and elusive. Consequently, distilling these into concrete, actionable insights for clinical use can be challenging. The richness of these experiences tends to resist straightforward categorization or simplification. Thus, practitioners may find it challenging to effectively utilize such complex and nuanced descriptions in their clinical practice. Therefore, formulating clinical implications alongside the results of the analysis can help guide practitioners’ understanding within a clinical context.

## Note

1. Lifeworld theory is part of phenomenology and continental philosophy, which also includes other theories, i.e., transcendental ideas, the epoché. However, the lifeworld theory has proven to be a solid approach to qualitative research even if the deeper philosophical notions are not included (K. Dahlberg et al., 2008; Zahavi, 2019, 2021).
